# Multiple Anterior Mitral Valve Perforation After Deep Transfemoral Aortic Valve Implantation

**DOI:** 10.21470/1678-9741-2020-0566

**Published:** 2022

**Authors:** Hakan Fotbolcu, Ramazan Özdemir

**Affiliations:** 1 Department of Cardiology, Bezmialem Vakıf University, Istanbul, Turkey.

**Keywords:** Transcatheter Aortic Valve Replacement, Heart Valve Prosthesis, Endocarditis, Bacterial, Mitral Valve, Sepsis

## Abstract

Transcatheter aortic valve implantation (TAVI) is an alternative for high-risk aortic valve replacement. There are limited data related to the late complications of TAVI. Deep aortic prosthetic valve implantation can cause direct erosive perforation of anterior mitral leaflet or erosive endothelial lesion which predisposes the tissue to infective endocarditis. Our report emphasizes anterior mitral leaflet perforation after TAVI, which may be seen especially in patients exposed to sepsis.

**Table:** 

Abbreviations, acronyms & symbols	
ID	= Implantation depth
IE	= Infective endocarditis
TAVI	= Transcatheter aortic valve implantation

## INTRODUCTION

Late complications of transcatheter aortic valve implantation (TAVI) are broadly unknown. In contrast to mitral valve laceration during antegrade transfemoral implantation, deep aortic prosthetic valve implantation can cause direct erosive perforation of the anterior mitral leaflet or erosive endothelial lesion which predisposes the endothelium to infective endocarditis. Information in the literature about the order of occurrence of perforation and infection is limited.

### Case Report

**Fig. 1:**
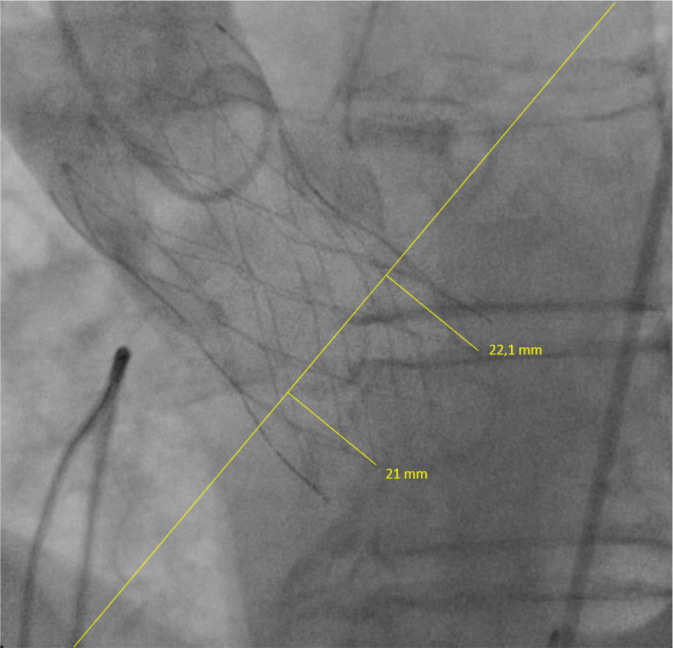
Postoperative aortogram (LAO 12.2°, CRAN 10.1°) with depth implantation metrics for non-coronary and left coronary cusps.

A 75-year-old male patient with severe aortic stenosis and severe renal failure was admitted to our hospital for a TAVI procedure. He successfully underwent implantation of a 29-mm Medtronic CoreValve Evolut R self-expandable aortic prosthesis via transfemoral access through the device implanted slightly deeper. Post-implantation aortogram showed mild aortic regurgitation. Implantation depth (ID) is defined as the distance between the aortic annular plane and the lower end of the prosthetic valve^[1]^. The ID of valve for the non-coronary cusp was 21 mm and for the left coronary cusp was 22.1 mm in LAO cranial view (LAO 12.2° and CRAN 10.1° - Figure 1). Patient’s echocardiographic outcomes included a mild aortic paravalvular leakage towards the septum (no contact with the mitral valve), low transvalvular gradient, mild mitral regurgitation, and normal ejection fraction on the 2^nd^ postoperative day. The patient was discharged three weeks later, with a jugular temporary catheter for dialysis since his kidney functions were completely lost.

**Fig. 2:**
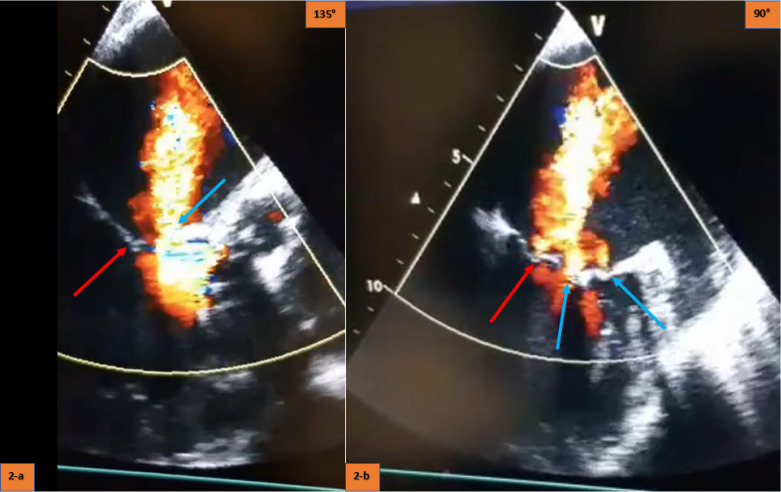
(A) Red arrow shows native mitral valve coaptation line; blue arrow shows anterior mitral valve perforation hole. (B) Red arrow shows native mitral valve coaptation line with mild degree regurgitation; blue arrows show two different holes from anterior mitral valve perforation.

The patient was readmitted to our hospital with febrile sepsis two months after surgery. Jugular catheter infection was detected and blood culture identified Staphylococcus aureus as the pathogen. Echocardiographic evaluation of the patient did not changed during this period and did not suggest any signs of mechanical complication. Appropriate antibiotic therapy was applied and blood cultures were negative for infection. A permanent dual-chamber pacemaker was implanted, since a complete heart block occurred in this period. Furthermore, a permanent dialysis catheter was placed in the left jugular vein for the maintenance of dialysis. The patient was discharged in excellent health conditions, however, he was readmitted again with symptoms of dyspnea. Examination of the patient revealed apical systolic murmur and rales in breath sounds. There was no sign of infection or indication of biochemical markers six months after surgery. Transesophageal echocardiography showed obvious friction between the anterior mitral valve and the distal stent frame of the valve prosthesis and two different perforation holes of the anterior mitral valve (Figure 2, Videos 1 and 2). The degree of mitral regurgitation was rated as severe. Non-surgical management was decided due to EuroSCORE II of the patient (10.7%). The patient is currently monitored under stable conditions with dialysis treatment three times a week, avoiding volume overload.

**Video 1:**
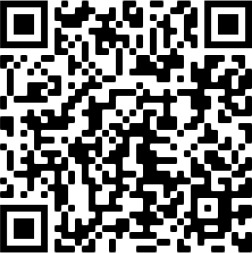
Obvious friction site between the anterior mitral valve and the distal stent frame of self-expandable prostheses with perforation hole and high degree mitral regurgitation.

**Video 2:**
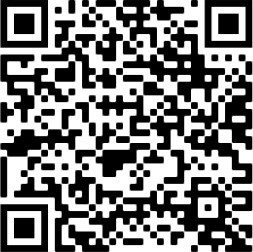
Native mitral valve coaptation line with mild regurgitation and two different perforation holes of the anterior mitral valve.

## DISCUSSION

Accurate device placement during transcatheter prosthetic valve implantation is significant for optimal results. Correct implantation was defined as a depth <6 mm below the annulus plane, and a depth >6 mm was considered as low implantation^[1]^. Some studies indicate that shallow ID in Medtronic Core-Valve System resulted in lower rates of new permanent pacemaker implantation^[1]^. Moreover, ID has also been detected as a predictor of paravalvular aortic regurgitation^[2]^. Our case shows that the precise measurement of the distance between non-coronary cusp and anterior mitral leaflet preoperatively and bearing in mind this distance when implanting the device in the catheter room can be important for avoiding this kind of complication, especially taking into account individual anatomical variations. In addition, slight changes in angles of angiographic positions can hamper accurate measurement of ID and lead to unsuitable deployment of the device.

So far, five similar cases have been published. Three of them reported mitral perforation as the underlying lesion, where infective endocarditis (IE) appears later on. The opposite chronology (IE causing mitral perforation) was suggested in the two other cases^[3^-^5]^. Our case included a duration related to sepsis, but mitral valve perforation without criteria for infection was detected six months later. The contribution of septicemia to the perforation process of anterior mitral valve as an erosive factor was not distinct. Besides, a paravalvular aortic leak had no contact with the mitral valve, resulting in endothelial erosion that predisposed to infective endocarditis. Our case contributes to the literature by revealing that deep transcatheter aortic prostheses implantation can lead to multiple perforations in the anterior mitral valve.

## CONCLUSION

Late mitral valve perforation should considered in patients with a history of TAVI and septicemia, complaints about dyspnea and admission to emergency room. In addition, operators should pay close attention to avoiding deep implantation of transcatheter aortic prostheses.

**Table:** 

Authors' roles & responsibilities	
HF	Substantial contributions to the conception or design of the work; or the acquisition, analysis or interpretation of data for the work; final approval of the version to be published
RÖ	Drafting the work or revising it critically for important intellectual content; final approval of the version to be published
